# Small vessel childhood primary angiitis of the central nervous system with positive anti-glial fibrillary acidic protein antibodies: a case report and review of literature

**DOI:** 10.1186/s12883-023-03093-x

**Published:** 2023-02-03

**Authors:** E Datyner, V Adeseye, K Porter, I Dryden, A Sarma, N Vu, AE Patrick, P Paueksakon

**Affiliations:** 1 Department of Pediatrics, Vanderbilt University Medical Center, Nashville, TN USA; 2 Vanderbilt University, Nashville, TN USA; 3 Department of Pathology, Microbiology and Immunology, Vanderbilt University Medical Center, 1161 21St Avenue South, Nashville, TN MCN C2318B37232-2561 USA; 4 Department of Radiology and Radiological Sciences, Vanderbilt University Medical Center, Nashville, TN USA

**Keywords:** Small vessel childhood primary angiitis of the central nervous system, Brain biopsy, Anti-glial fibrillary acidic protein antibodies

## Abstract

**Background:**

Small vessel childhood primary angiitis of the central nervous system (SV-cPACNS) is a rare disease characterized by inflammation within small vessels such as arterioles or capillaries.

**Case presentation:**

We report a case of SV-cPACNS in an 8-year-old boy confirmed by brain biopsy. This patient was also incidentally found to have anti-glial fibrillary acidic protein (GFAP) antibodies in the cerebrospinal fluid (CSF) but had no evidence of antibody-mediated disease on brain biopsy. A literature review highlighted the rarity of SV-cPACNS and found no prior reports of CSF GFAP-associated SV-cPACNS in the pediatric age group.

**Conclusion:**

We present the first case of biopsy proven SV-cPACNS vasculitis associated with an incidental finding of CSF GFAP antibodies. The GFAP antibodies are likely a clinically insignificant bystander in this case and possibly in other diseases with CNS inflammation. Further research is needed to determine the clinical significance of newer CSF autoantibodies such as anti-GFAP before they are used for medical decision-making in pediatrics.

## Introduction

Central nervous system (CNS) vasculitis is a disease characterized by inflammation of cerebral or spinal blood vessels. It can be a primary disorder, or more commonly a secondary disorder due to systemic illness [1, 2]. Childhood primary angiitis of the central nervous system (cPACNS) is a rare disease in which patients have neurologic deficits with or without accompanying angiographic abnormalities [3]. Angiography is unable to show inflammation within small vessels such as arterioles or capillaries. Thus, in small vessel cPACNS (SV-cPACNS), the diagnosis must be confirmed with a brain biopsy [4]. Other inflammatory brain diseases, such as those mediated by autoantibodies, are often clinically indistinguishable from SV-cPACNS [5]. Glial fibrillary acidic protein (GFAP) astrocytopathy is a newly discovered disease mediated by IgG binding to GFAP, a protein within the CNS [6–8]. We present a case of biopsy proven, small vessel cPACNS in a pediatric patient. Incidentally, this patient was found to have GFAP CSF antibodies, but had no evidence of antibody-mediated disease on brain biopsy. To our knowledge there are no published cases of pediatric cPACNS associated with positive GFAP CSF antibodies. We will use this case to review SV-cPACNS and discuss GFAP CNS antibodies as a non-pathologic marker of CNS inflammation rather than as a pathologic autoantibody that causes disease.

## Case presentation

An 8-year-old incompletely immunized boy with a history of mild speech delay presented to the emergency department with 1 week of fever, headache, fatigue, unsteady gait, abdominal pain and vomiting. An outpatient evaluation 2 days prior included negative tests for strep, influenza, and SARS-COV-2. Immediate household family members were negative for SARS-COV-2. On arrival to the emergency department, the patient was ill appearing, lethargic, tachycardic, and febrile to 104.4F. Physical examination was notable for nuchal rigidity.

Initial labs showed a mild leukopenia (WBC 4.6 × 10^3/mcl), CSF pleocytosis (190 cells/mcL, 95% lymphocytes) and elevated CSF protein (132 mg/dL) (Table 1). Inflammatory markers (CRP, ESR) were normal. With high suspicion for viral, rickettsial, or atypical bacterial meningoencephalitis, a broad infectious work up and empiric antimicrobial treatment were initiated.

**Table 1:** Serial Cerebrospinal Fluid Studies

Serial Cerebrospinal Fluid Studies *(abnormal values in bold)*				
Lumbar Puncture	1st	2nd	3rd	4th
WBC (cells/mcl)	**190 (95% lymphocytes)**	**145 (89% lymphocytes)**	**263 (84% lymphocytes)**	**131 (76% lymphocytes)**
RBC (cells/mcl)	89	1	15	2
Protein (mg/dL)	**132**	**235**	**245**	**119**
Glucose (mg/dL)	**37**	**38**	**31**	**40**

The following day, he developed bilateral ankle clonus, hyperreflexia, and dysmetria. MRI brain was normal**.** Repeat lumbar puncture showed a continued CSF lymphocytic pleocytosis (145 cells/mcL) and worsened elevated protein (235 mg/dL). Mental status and neurologic examination continued to rapidly decline and by day 3 of admission he was less responsive with new findings of nystagmus, urinary incontinence, and dysconjugate gaze with right sided esotropia. EEG showed no signs of seizure or status epilepticus. Due to his worsening neurologic status despite broad-spectrum antimicrobial therapy, there was heightened concern for an autoimmune, paraneoplastic, or malignant etiology. Antimicrobial coverage was discontinued as infectious studies returned normal. The infectious work up was negative except for a positive mycoplasma IgG and IgM that was treated with a full course of levofloxacin (Table 2). Hematology-oncology was consulted and ruled out malignancy. Serum and CSF flow cytometry as well as bone marrow biopsy were normal.

**Table 2:** CSF and Serum Infectious studies *(positive results in bold)*

CSF	Serum
Bacterial, fungal, AFB Culture EBV PCR HSV-1/2 PCR Ehrlichia/anaplasma PCR Cryptococcal antigen Histoplasma antibody Lymphocytic choriomeningitis virus serology Arbovirus antibody panel Mycoplasma pneumoniae PCR Biofire meningoencephalitis PCR panel Cryptosporidum antibody Borrelia (lyme) PCR Mycobacterium Tuberculosis (MTB) complex PCR UCSF metagenomics panel	Bacterial Culture EBV PCR and serology HSV-1/2 PCR and serology Ehrlichia/anaplasma PCR and serology Cryptococcal antigen Histoplasma Antigen Lymphocytic choriomeningitis virus serology Arbovirus antibody panel **Mycoplasma Pneumoniae serology (IgG and IgM)** CMV PCR and serology Hepatitis B core antibody and surface antigen HIV p24 and HIV 1/2 antibody Bartonella serology Brucella serology Rocky Mountain Spotted fever serology Coccidiodes serology Blastomyces serology Rabies serology Leptospirosis serology COVID-19 PCR and serology

**Fig. 1:**
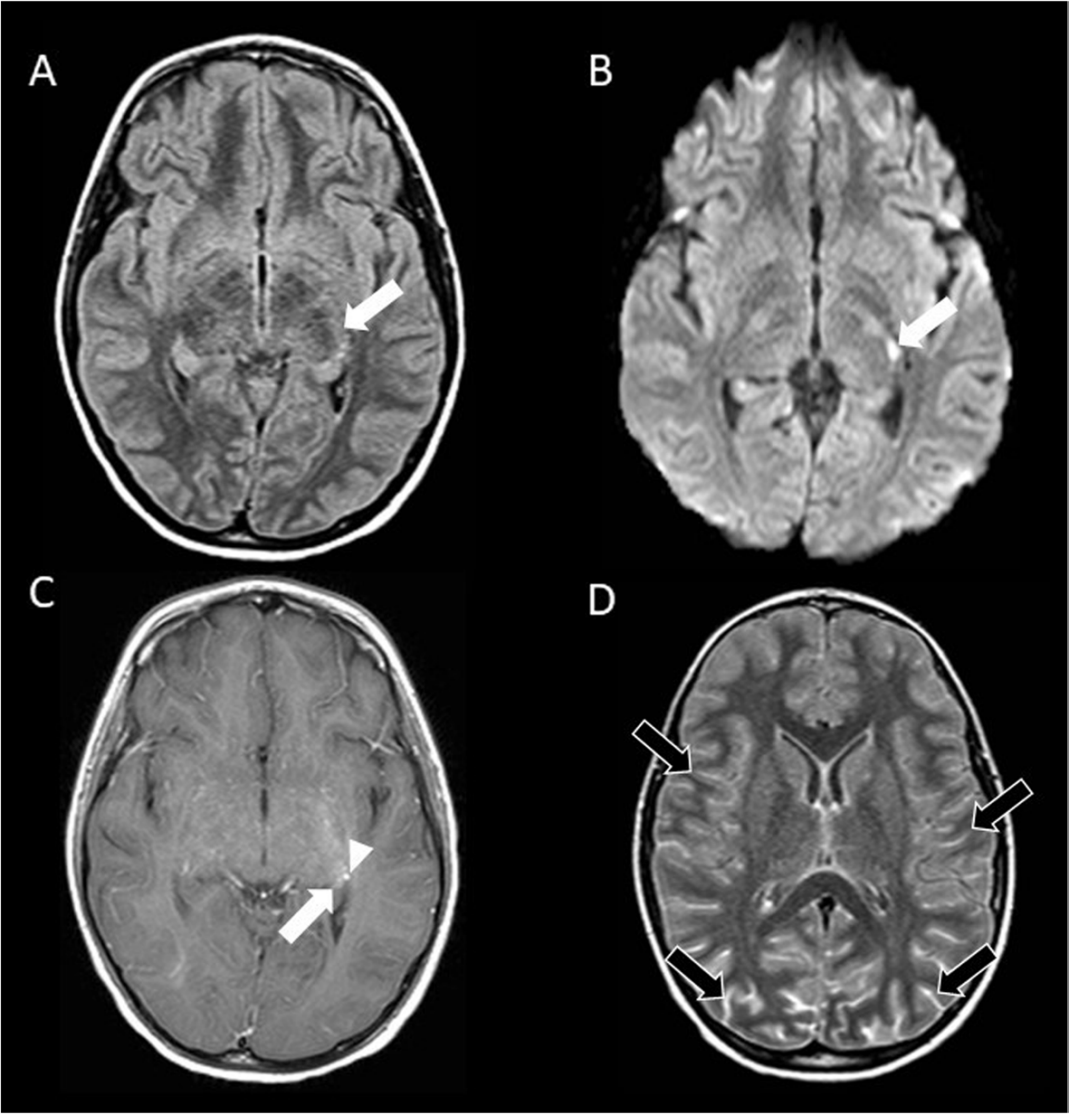
MRI of the brain obtained on day 5 of admission. **A**-**C**. Axial pre-contrast FLAIR (**A**), DWI (**B**) and post-contrast T1-weighted (**C**) MRI demonstrate a small FLAIR hyperintense lesion (white arrows) with low diffusivity and faint enhancement in the left thalamus (noting incidental adjacent developmental venous anomaly, arrowhead in C). **D**. Axial postcontrast FLAIR demonstrates diffuse leptomeningeal enhancement (black arrows) that was not apparent on the post-contrast T1-weighted images

Repeated brain MRI on day 5 showed new findings of pronounced leptomeningeal hyperintensity and focal low diffusivity in the region of the dorsal left thalamus (Fig. 1). Within a day, the patient was transferred to the pediatric intensive care unit due to autonomic instability, decreased responsiveness, and abnormal eye movements concerning for seizures. Antiepileptics were given and produced no improvement in mental status.

Rheumatology was consulted and considered a differential of autoimmune encephalitis, CNS vasculitis, acute disseminated encephalomyelitis (ADEM), CNS sarcoidosis, and CNS lupus. All rheumatologic labs returned normal, and the Mayo encephalopathy autoimmune panel was sent and not yet resulted (Table 3). Testicular ultrasound, thyroid ultrasound, and ophthalmologic examination were normal. A third lumbar puncture showed worsening CSF pleocytosis (263 cells/mcL) and protein (245 mg/dL) (Table 1). Rheumatology recommended pursuing brain biopsy if all oncologic and infectious testing remained negative.

**Table 3:** CSF and Serum Rheumatologic Studies *(positive results in bold)*

CSF	Serum
Anti-NMDA receptor IgG CSF oligodendrocyte or MOG antibody Mayo CSF paraneoplastic auto antibody panel **Mayo encephalopathy autoimmune panel**	ANA with reflex ENA/DNA C3, C4 levels ACE level Lysozyme Level ANCA antibody panel sCD163 (Cinncinati) Neopterin (Cinncinati) IL-18 (Cinncinati) **Cytokine Panel (Cinncinati), High Il-6** CXCL9 (Cinncinati) Soluble IL-2R (Cinncinati)

**Fig. 2:**
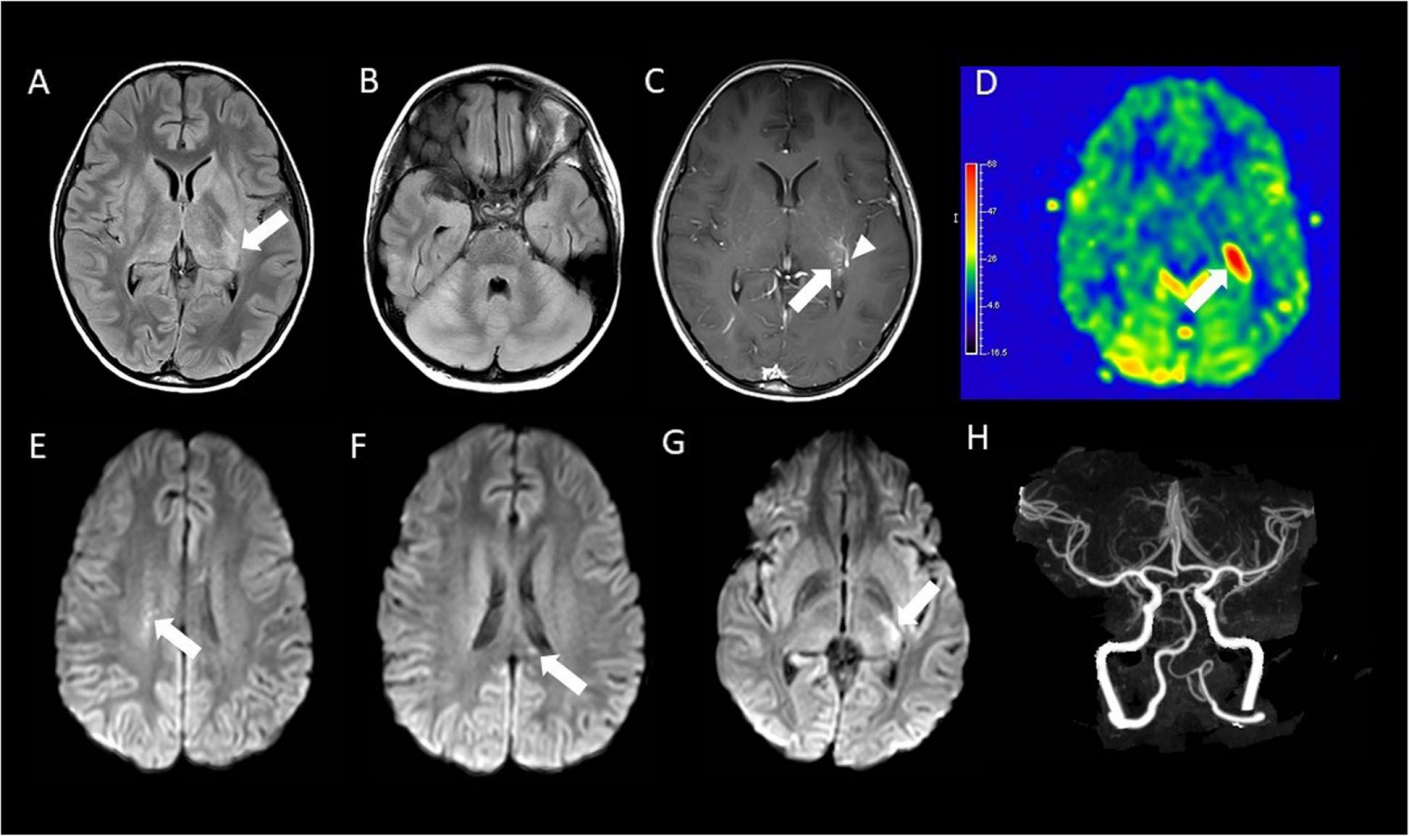
MRI of the brain obtained on day 11 of admission and CT angiogram of the head obtained on day 13 of admission. **A** and **B**. Axial pre-contrast FLAIR demonstrates increased conspicuity of the original focal thalamic lesion (arrow in A), as well as new, extensive abnormal FLAIR hyperintensity within the basal ganglia, thalami, cortex, brainstem, and cerebellum, with effacement of the cerebellar sulci **C**. Axial postcontrast T1-weighted imaging demonstrates increased enhancement of the lesion (arrow; noting incidental adjacent developmental venous anomaly, arrowhead). **D**. Axial arterial spin labeling image shows increased perfusion of the thalamic lesion. **E**–**G**. DWI images from superior (**E**) to inferior (**G**) demonstrate new lesions (arrows) in the right centrum semiovale (**E**) and splenium of corpus callosum (**F**), as well as increased size of the original focal thalamic lesion (**G**). **H**. Coronal maximum intensity projection image from a CT arteriogram of the head is normal

Prior to performing a brain biopsy, the multidisciplinary team decided to treat empirically for an autoimmune CNS process with 2 doses of IVIG and a 5-day course of plasmapheresis. Steroids were considered but not initiated until all oncologic and infectious etiologies could be ruled out. The patient had no clinical neurologic improvement, and there was a slight improvement in CSF pleocytosis and protein (Table 1). A repeat brain MRI showed progression of diffuse parenchymal swelling involving the cortex, deep gray nuclei, hypothalamus, brainstem, and cerebellum. The size of a focus of low diffusivity in the dorsal thalamus, with T2 prolongation, faint enhancement, and hyperperfusion evident on arterial spin labeling was increased. A new small foci of low diffusivity in the centrum semiovale and callosal splenium appeared (Fig. 2). Importantly, cerebral CT angiography was normal, ruling out medium or large vessel vasculitis.

Up to this point, the patient had an extensive negative work up and no response to treatment. Brain biopsy was deemed necessary to rule out small vessel vasculitis and isolated CNS hemophagocytic lymphohistiocytosis (HLH) before initiating empiric treatment with steroids. Brain tissue was obtained, and dexamethasone initiated. The following day, the preliminary pathology diagnosis was consistent with small vessel CNS vasculitis.

**Fig. 3:**
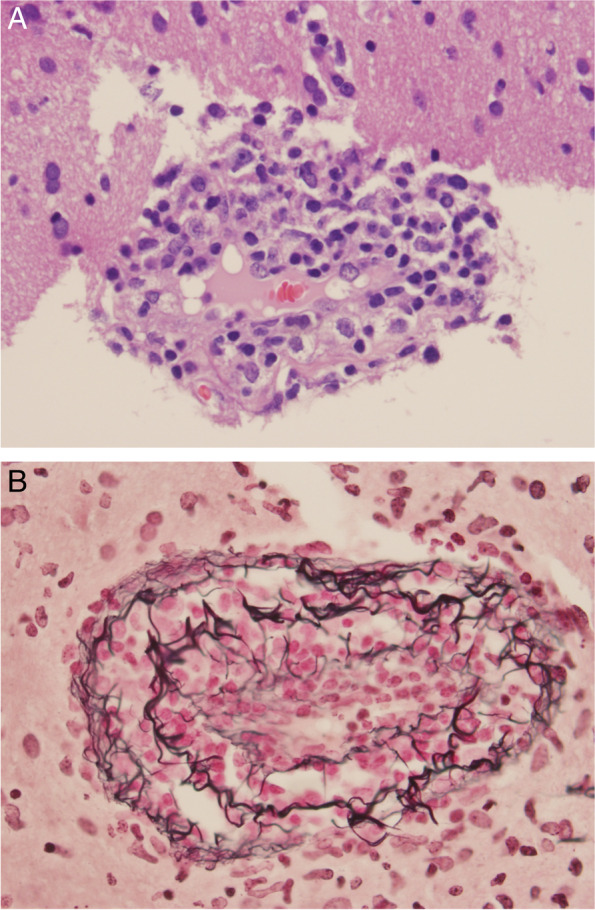
**A** and **B**: Vasculocentric transmural inflammation with damage to the vascular wall of arteries without fibrinoid mural necrosis (**A**, Hematoxylin and Eosin stain, × 400 and **B**, Reticulin stain, × 200)

**Fig. 4:**
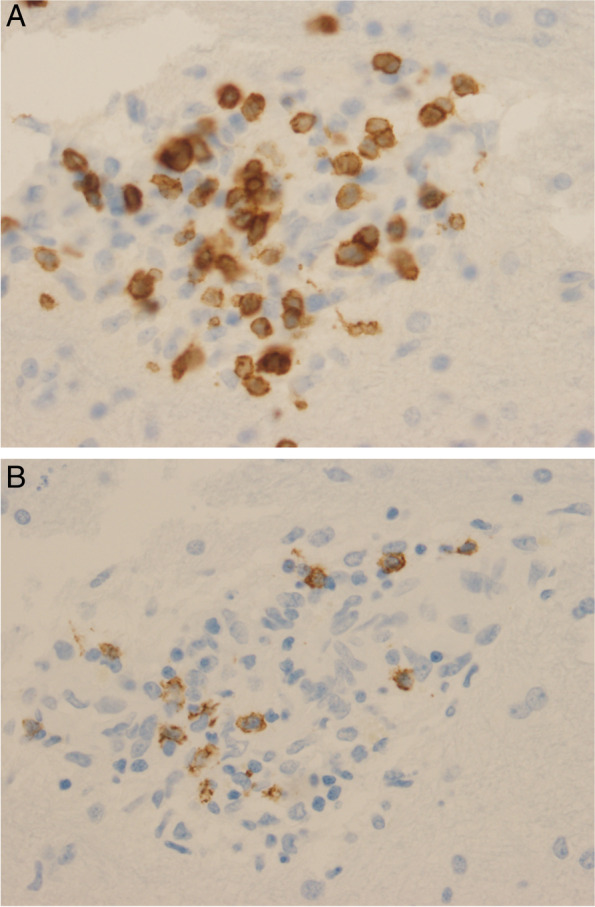
**A** and **B**: The inflammatory infiltrate is composed mainly of lymphocytes which is highlighted by immunohistochemical stains of CD3 (A, anti-CD3, × 400) and CD20 (B, anti-CD20, × 400) as markers of T and B lymphocytes

The histopathologic assessment showed vasculocentric transmural inflammation with damage to the vascular wall of arterioles, capillaries, and extent venules, without fibrinoid mural necrosis (Figs. 3A and 3B). The inflammatory infiltrate was composed mainly of lymphocytes as highlighted by immunohistochemical stains of CD3 and CD20 as markers of T and B lymphocytes (Figs. 4A and 4B). There were no granulomas. A Congo red stain was negative for amyloid. There was reactive gliosis of brain parenchyma, which was highlighted by GFAP stain. These findings were diagnostic of SV-cPACNS.

Dexamethasone was switched to high dose intravenous methylprednisolone for 5 days followed by a prolonged oral taper over 12 months with appropriate pneumocystis pneumonia and gastric prophylaxis, per the Brainworks treatment protocol for SV-cPACNS. One week after steroid initiation, the patient began following simple motor commands. Induction immunosuppressive therapy with cyclophosphamide was started on the same day. After another week, he was interactive, speaking in short sentences, and eating a soft diet. Prior to discharge, the Mayo CSF autoimmune encephalitis panel returned with CSF GFAP antibody positivity. Given the patient’s tissue diagnosis of small vessel vasculitis and dramatic improvement with immunosuppressive therapy, management was not changed. Three weeks after initiation of steroids and two weeks after initiation of cyclophosphamide, he was discharged home with outpatient therapy.

**Fig. 5:**
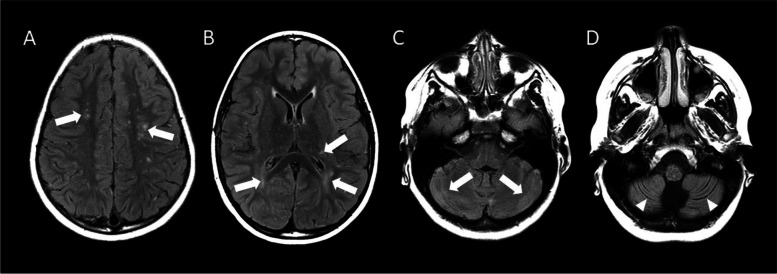
Axial FLAIR images obtained 3 months after hospitalization. **A**-**D**. Sequential FLAIR MRI from superior (**A**) to inferior (**D**) demonstrate small hyperintense lesions within the centrum semiovale (arrows; **A**) and periventricular white matter and left thalamus (arrows; **B)** that are more numerous than on the previous examination. Signal abnormality in the basal ganglia and thalamus is substantially improved. Abnormal hyperintense signal presumably representing gliosis (arrows; **C**) and fissural prominence denoting volume loss (arrowheads; **D**) are seen in the cerebellum

After completing 7 monthly doses of cyclophosphamide as an outpatient, the patient was transitioned to oral mycophenolate mofetil for maintenance immunosuppression. At his 6 month follow up, he continued to have intermittent slow speech and difficulty with cognitive processing. A follow up MRI 3 months later showed resolution of the acute appearing-appearing diffuse parenchymal abnormalities, with numerous new white matter lesions consistent with microvascular ischemic disease and cerebellar volume loss (Fig. 5).

## Discussion

We have presented a case of SV-cPACNS in an 8-year-old boy that resulted in severe neurologic dysfunction. This is the first reported case of SV-cPACNS with associated positive GFAP CSF antibodies.

The three known types of cPACNS are differentiated by findings on angiography and progression of disease. These include: angiography positive (medium or large vessel) progressive (P-cPACNS), angiography positive (medium or large vessel) non-progressive (NP-cPACNS) and angiography negative (small vessel) (SV-cPACNS) [3]. cPACNS diagnostic criteria were originally proposed in the adult literature by Calabrese et al. [9].

- 1. The presence of an unexplained neurologic deficit after thorough clinical and laboratory evaluation
- 2. Documentation by cerebral angiography and or tissue examination of an arteritic process within the central nervous system
- 3. No evidence of a systemic vasculitis or any other condition to which the angiographic or pathologic features could be secondary

cPACNS is rare and the true incidence is unknown [3]. Secondary vasculitis due to an infectious, autoimmune, neoplastic, or drug-induced cause, is much more common [2]. Non-vascular mimics of SV-cPACNS include demyelinating disease, Hashimoto encephalitis, infectious encephalitis, metabolic disease, neoplastic disease, nutritional deficiency, and other inflammatory brain disease [10].

The differential for this patient’s initial presentation was most concerning for an infectious process and other high considerations on the differential included a parainfectious/post infectious inflammatory demyelinating entity such as MOGAD-ADEM, ADEM, pediatric onset Multiple Sclerosis (MS), or an inflammatory astrocytopathy like NMOSD-AQP4 + . Common symptoms of an autoimmune demyelinating processes include signs of optic neuritis, transverse myelitis, brainstem-cerebellar involvement, and multifocal neurologic deficits. Detailed clinical history, neurologic exam and imaging study results are crucial for guiding the initial workup. Pediatric onset MS is rare with approximately 5–10% of cases having symptoms prior to 18 years old and less than 5% having symptoms prior to 10 years old [11]. MS is a clinical diagnosis requiring dissemination in space and time by the McDonald Criteria, and supportive paraclinical findings include MRI, CSF, and ophthalmologic assessments. MOGAD is now recognized as an antibody-mediated demyelinating disease that is distinct from multiple sclerosis and has overlapping features with NMOSD. MOGAD encompasses broad clinical phenotypes including ADEM, severe bilateral optic neuritis that is very sensitive to steroids, myelitis that often involves the lower thoracic and lumbar regions, cortical encephalitis with seizures and behavioral changes with focal FLAIR-abnormality on brain MRI accompanied by leptomeningeal enhancement (FLARE and FUEL, respectively), brainstem encephalitis, and/or a combination of these. The MOGAD clinical phenotype changes with age in the pediatric population, with patient older than 9 years old exhibiting a more optico-spinal presentation and younger patients exhibiting more commonly an ADEM presentation [12]. NMOSD-AQP4 + is an astrocytopathy with high morbidity and mortality from severe attacks. NMOSD- AQP4 + clinical manifestations include severe optic neuritis (longitudinally extensive involvement of either unilateral or bilateral optic nerves often with chiasmal involvement), longitudinally extensive transverse myelitis, area postrema syndrome with intractable nausea/emesis, diencephalic involvement with altered mental status and endocrine disturbances, and hemispheric syndrome. The CSF and MRI findings of demyelinating diseases are compared in Table 4.

**Table 4:** Comparison of findings in vasculitis and demyelinating diseases

	Multiple Sclerosis	MOGAD	NMOSD- + AQP4	Vasculitis
CSF cell count	Normal-mild pleocytosis < 50cells/uL	Pleocytosis: 31- > 100cells/uL [13]	Pleocytosis: mild to > 50cells/uL range (6–380) [14]	Pleocytosis to variable degree
CSF cell differential	Lymphocytes predominance	Mostly lymphocytes and monocytes	Can include neutrophils, lymphocytes [14]	Usually lymphomonocytic dominance [15]
CSF protein	Normal to slightly elevated	Normal to mildly elevated	Normal to mildly elevated	Mild to moderate elevation
Oligoclonal band	> 90%	Rare (< 10%) [13]	rare	rare
IgG index	Usually elevated	Usually elevated	Usually elevated	rare
Brain MRI characteristic findings	Characteristic round, ovoid lesions > 5 mm in periventicular, juxtacortical, brainstem and cerebellar involvement (peduncles)	Variable- no specific characteristics: usually multifocal subcortical WM, deep grey matter, brainstem involvement; compared to NMO-AQP4 + presence of cortical/subcortical WM distinguish; area postrema involvement is rare [16]; FLARE/FUEL (focal FLAIR-abnormality on brain MRI accompanied by leptomeningeal enhancement)	Typically follows AQP4 distribution: hypothalamic, brainstem periependymal, lesion of area postrema and splenium lesion [17]	Variable: multifocal parenchymal ischemic change, can have small area of hemorrhage, can have meningeal enhancement
Histologic findings	Perivenular lymphocytic cuffing and white matter degeneration	Perivenular lymphocytic cuffing and confluent white matter degeneration	Astrocyte lysis followed by infiltration of neutrophils in brain parenchyma and reactive gliosis	Transmural lymphocytic infiltrate with damage to the vascular wall (with or without fibrinoid necrosis) including arterioles, capillaries, and to a lesser extent, venules

Our patient’s broad diagnostic work up for secondary etiologies was negative except for positive serum mycoplasma IgM and IgG. Mycoplasma pneumonia can cause large and medium vessel CNS vasculitis on angiography, leading to stroke symptoms [18–20]. However, we found no published cases of small vessel CNS vasculitis associated with mycoplasma. We do not believe this was the cause of the clinical presentation in our case due to the lack of respiratory symptoms, negative mycoplasma PCR in CSF and respiratory secretions, and lack of clinical improvement after a course of levofloxacin.

SV-cPACNS presents with predominantly diffuse neurologic deficits, mild to moderately elevated inflammatory markers, multifocal MRI lesions, and normal angiography. The diagnosis is confirmed by a brain biopsy showing intramural lymphocytic infiltration of small cerebral arteries [1–4, 21]. In contrast, angiography positive cPACNS presents with focal neurologic findings, unilateral or multifocal MRI lesions. Diagnosis is confirmed by vessel stenosis on cerebral angiography [3, 22]. Other reported cases of SV-cPACNS demonstrated similar presenting symptoms as our case, with the most common being seizure (85%), headache (62%), systemic symptoms (flu-like symptoms, fever, abdominal pain, fatigue; 75%), and cognitive decline leading to poor school performance (50%) [2, 4]. More severe neurologic symptoms such as acute loss of consciousness and refractory meningoencephalitis, like those in our patient, result most commonly from extensive meningeal and adjacent cortical involvement [3].

A treatment protocol for SV-cPACNS published by Beelen et al. [23] consists of an induction phase with IV methylprednisolone followed by an oral prednisone taper for 12 months and monthly cyclophosphamide infusions for 6 months. A maintenance phase with 18 months of mycophenolate mofetil or mycophenolic acid begins after completion of the cyclophosphamide. When treated with similar protocols, the majority of patients have good neurologic outcomes [21, 22]. However, Cellucci et al. [22] found that among patients with all 3 types of cPACNS, diagnosis of SV-cPACNS and seizures at presentation were early risk factors for persistently higher disease activity.

Other inflammatory brain diseases are often indistinguishable from SV-cPACNS by clinical, laboratory, and imaging evaluation [5]. In SV-cPACNS, markers of systemic inflammation can be normal to moderately elevated and there is no specific CSF autoantibody pattern [1–4, 21]. Brain MRI is highly sensitive for lesions in cPACNS; however, imaging findings are often non-specific and can even be normal [4, 5]. Interestingly, in our case radiographic findings lagged behind emerging clinical signs and MRI was normal until day 5 of hospitalization. Objective measures such as CSF pleocytosis, elevated CSF protein, and elevated opening pressure on LP are more likely in SV-cPACNS than P-cPACNS or NP-cPACNS, but are not specific when differentiating SV-cPACNS from other inflammatory brain diseases [3]. A brain biopsy is required for definitive diagnosis of SV-cPACNS [5, 24]. Brain biopsy has an important role in excluding infectious, malignant, and demyelinating diagnoses [24]. In our case, the brain biopsy revealed the pathologic immune mechanisms at play, allowing a more targeted treatment strategy. It is important to consider brain biopsy early and delay steroids until tissue is obtained because pre-biopsy steroid treatment and prolonged time to biopsy are correlated with inconclusive biopsies that lack diagnostic features of SV-cPACNS [4].

The diagnosis of vasculitis on biopsy requires angiocentric transmural inflammation with damage to the vascular wall (with or without fibrinoid necrosis). In SV-cPACNS this usually involves the small leptomeningeal and parenchymal vessels including arterioles, capillaries, and to a lesser extent, venules. Three patterns of destructive vasculitis are characterized: granulomatous, lymphocytic, and necrotizing. Lymphocytic vasculitis, as seen in this case, is the second most common pattern [25]. The inflammatory infiltrate is composed of lymphocytes with occasional plasma cells, in multiple layers, extending through the vascular wall, causing transmural inflammation with vascular distortion and destruction. For the definitive diagnosis of lymphocytic vasculitis, marked lymphocytic vascular infiltrate in the absence of significant parenchymal inflammation is required. Additional immunohistochemical stains to identify CD3 and CD20 are useful to determine T versus B cell mediated disease, respectively. The CSF and MRI findings of vasculitis and demyelinating diseases are compared in Table 4.

Neuronal antibody mediated brain disease is a compelling mimic of SV-cPACNS. GFAP astrocytopathy, a neuronal antibody mediated encephalitis syndrome mediated by CSF GFAP antibodies, was first described in 2016 [7, 8]. It is a meningoencephalomyelitis with symptoms of fever, headache, encephalopathy, myelitis, and abnormal vision [6, 7]. Although multiple adult cases have been published, there are very few pediatric cases in the literature (Table 5) [7, 26–37].

**Table 5:** Summary of clinical characteristic, clinical outcome, CSF findings, and treatment in published pediatric patients

Publication	Number of cases and ages	Clinical Characteristics	CSF pleocytosis range (cells/mcl)	Positive Antibodies	Treatment	Brain biopsy performed	Vasculitis noted on biopsy	Clinical outcome
**Pediatric Cases**								
Dubey et al. 2018 [31]	10 children, 3–15 yo	Viral prodrome, ataxia, seizures meningoencephalitis, peripheral neuropathy, autonomic dysfunction	12–159	GFAP in serum and CSF	Steroids IVIG Plasmapheresis Rituximab	No	-	5/10 – response to 1^st^ line therapy 2/10—response to 2^nd^ line therapy Outcome not indicated for 3 pts
Francisco et al. 2019 [32]	One 6 yo child	Meningoencephalitis, abnormal eye movements, dysautonomia	198, 385	GFAP CSF Mycoplasma IgG, IgM	Steroids IVIG Rituximab	No	-	Normal neuro exam 17 months after initial symptoms, attention problems at school
Handoko et al. 2019 [33]	One 12 yo child	Acute psychiatric decline 12 mo. after herpes simplex meningitis with migraines, memory deficits, impulsivity, hypersexuality, hallucinations	“Normal”	GFAP serum and CSF	Steroids IVIG Mycophenolate mofetil	No	-	Waxing and waning neuropsychiatric symptoms, cognitive difficulties in school
Martin et al. 2018 [30]	One 13 yo child	Meningoencephalitis, photophobia, phonophobia, agitation, ataxia, hyperreflexia, dysautonomia	204	GFAP CSF NMDA-R CSF	Steroids IVIG Plasmapheresis	No	-	Ovarian teratoma identified and removed. Drastic improvement in neurologic symptoms after 2 months. Short term memory difficulty
Oger et al. 2020 [34]	Two children, 10 and 16 yo	Meningoencephalitis, gait difficulty, nystagmus, dysautonomia, bladder dysfunction	280–700	GFAP serum and CSF	Steroids	No	-	Complete Recovery with normal brain MRI
Rutatangwa et al. 2020 [35]	One 6 yo child	Meningoencephalitis	198, 385	GFAP CSF	Steroids IVIG Rituximab	No	-	Significant improvement with some residual symptoms at 17 mo. follow up
Theroux et al. 2018 [36]	One 15 yo child	Meningoencephalitis, bilateral leg weakness, dizziness, catatonia, dysautonomia	100	GFAP CSF	IVIG Plasmapheresis Rituximab	No	-	Difficulty with memory and stamina, independent in all ADLS
Trau and Gallentine 2018 [37]	One 10 yo child	Meningoencephalitis, weakness, ascending paralysis	“Elevated”	GFAP serum	Steroids IVIG Plasmapheresis	No	-	Full recovery within one year

Dubey et al. [31], described the largest pediatric cohort consisting of 10 patients, ages 3–15, with serum or CSF GFAP antibody positivity. These children had symptoms including headache, neck stiffness, rhinorrhea, fever, cough, ataxia, and autonomic dysfunction [31]. Almost all of these symptoms were demonstrated in our case. Brain biopsies were not reported in any case of pediatric GFAP astrocytopathy on our literature review. In the small number of brain biopsies performed in adult patients with GFAP astrocytopathy, histopathology demonstrated lymphocytic perivascular inflammation, a distinctly different inflammatory pattern from that of true vasculitis [26–29, 38]. In our case, direct immunofluorescence studies also showed no immunoglobulin or complement, arguing against antibody mediated disease. Based on the objective evidence identified on brain biopsy, we determined that our patient’s GFAP CSF antibody positivity was a clinically insignificant bystander rather than a pathologic mechanism of disease. GFAP CSF antibodies have been found in the setting of diverse neoplasms, HSV infection, and other neurologic autoimmune diseases such as neuromyelitis optica and anti-N-methyl D- (NMDA) receptor encephalitis [7, 26, 28]. This supports that GFAP CSF antibodies can be non-pathologic and a bystander in disease processes with extensive CNS inflammation. We hypothesize that the exposure of GFAP intermediate filaments during CNS inflammation and destruction can lead to self-antigens the ultimately result in circulating GFAP auto-antibodies. As pediatric CSF antibody testing continues to expand and becomes more universal, further investigations will be necessary to determine the diagnostic significance of specific CSF antibodies to better guide clinical decision making in cases of pediatric meningoencephalitis.

In conclusion, we present the first case of biopsy proven SV-cPACNS vasculitis with associated positive GFAP CSF antibodies. Despite this positive antibody, we are confident that our case does not represent GFAP astrocytopathy based on our objective histopathologic data. In patients with systemic symptoms, diffuse neurologic dysfunction, and laboratory and imaging findings concerning for CNS inflammation, it is of utmost importance to pursue brain biopsy when an extensive evaluation for infectious, malignant, and systemic inflammatory conditions has been negative and SV-cPACNS is in the differential diagnosis [2, 3]. Our case illustrates that this is true in pediatric patients with positive CSF auto-antibodies. Pediatric brain biopsy can diagnose SV-cPACNS and rule out other mimicking conditions, especially inflammatory brain diseases such as GFAP astrocytopathy [1, 3, 4, 24, 39, 40, 41]. Histopathologic data from a biopsy is valuable for guiding targeted immunotherapy. This case demonstrates the previously unreported finding that GFAP CSF antibodies can be present but not pathologic in a patient with biopsy confirmed SV-cPACNS. We hypothesize that GFAP CSF antibodies may be clinically insignificant bystanders in diseases with marked CNS inflammation. Further research is needed to determine the clinical significance of newer CSF autoantibodies such as anti-GFAP, before they are used in medical decision-making in pediatrics.

## Data Availability

Not applicable.

## Abbreviations

cPACNS: Childhood primary angiitis of the central nervous system
SV-cPACNS: Small vessel childhood primary angiitis of the central nervous system
P-cPACNS: Progressive childhood primary angiitis of the central nervous system
NP-cPACNS: Non-progressive childhood primary angiitis of the central nervous system
MS: Multiple sclerosis
NMOSD: Neuromyelitis optica spectrum disorder
MOGAD: Myelin oligodendrocyte glycoprotein antibody –associated disease
ADEM: Acute disseminated encephalomyelitis
MOGAD-ADEM: Myelin oligodendrocyte glycoprotein antibody-associated disease with acute disseminated encephalomyelitis-like presentation
NMOSD-AQP4 +: Neuromyelitis optica spectrum disorder Aquaporin-4 antibody positive
GFAP: Glial fibrillary acidic protein
